# The Chinchilla Research Resource Database: resource for an otolaryngology disease model

**DOI:** 10.1093/database/baw073

**Published:** 2016-05-12

**Authors:** Mary Shimoyama, Jennifer R. Smith, Jeff De Pons, Marek Tutaj, Pawjai Khampang, Wenzhou Hong, Christy B. Erbe, Garth D. Ehrlich, Lauren O. Bakaletz, Joseph E. Kerschner

**Affiliations:** ^1^Rat Genome Database, Department of Surgery, Medical College of Wisconsin, Milwaukee, WI, USA; ^2^Department of Otolaryngology and Communication Sciences, Medical College of Wisconsin, Children's Hospital of Wisconsin, Milwaukee, WI, USA; ^3^Department of Microbiology and Immunology; ^4^Department of Otolaryngology-Head and Neck Surgery, Center for Genomic Sciences and Center for Advanced Microbial Processing, Institute of Molecular Medicine and Infectious Diseases, Drexel University College of Medicine, Philadelphia, PA, USA; ^5^Center for Microbial Pathogenesis, the Research Institute at Nationwide Children's Hospital and the Ohio State University College of Medicine, Columbus, OH, USA; ^6^Division of Pediatric Otolaryngology, Medical College of Wisconsin, Children's Hospital of Wisconsin, Milwaukee, WI, USA

## Abstract

The long-tailed chinchilla (*Chinchilla lanigera*) is an established animal model for diseases of the inner and middle ear, among others. In particular, chinchilla is commonly used to study diseases involving viral and bacterial pathogens and polymicrobial infections of the upper respiratory tract and the ear, such as otitis media. The value of the chinchilla as a model for human diseases prompted the sequencing of its genome in 2012 and the more recent development of the Chinchilla Research Resource Database (http://crrd.mcw.edu) to provide investigators with easy access to relevant datasets and software tools to enhance their research. The Chinchilla Research Resource Database contains a complete catalog of genes for chinchilla and, for comparative purposes, human. Chinchilla genes can be viewed in the context of their genomic scaffold positions using the JBrowse genome browser. In contrast to the corresponding records at NCBI, individual gene reports at CRRD include functional annotations for Disease, Gene Ontology (GO) Biological Process, GO Molecular Function, GO Cellular Component and Pathway assigned to chinchilla genes based on annotations from the corresponding human orthologs. Data can be retrieved via keyword and gene-specific searches. Lists of genes with similar functional attributes can be assembled by leveraging the hierarchical structure of the Disease, GO and Pathway vocabularies through the Ontology Search and Browser tool. Such lists can then be further analyzed for commonalities using the Gene Annotator (GA) Tool. All data in the Chinchilla Research Resource Database is freely accessible and downloadable via the CRRD FTP site or using the download functions available in the search and analysis tools. The Chinchilla Research Resource Database is a rich resource for researchers using, or considering the use of, chinchilla as a model for human disease.

**Database URL**: http://crrd.mcw.edu

## Introduction

### Disease model

The long-tailed chinchilla (*Chinchilla lanigera*) is used as a model for understanding the physiology, development and function of the auditory system. The chinchilla is used in several research areas, including otitis media, upper respiratory tract infections, hearing, psychoacoustics and ototoxicity. The chinchilla has been used for auditory or acoustics research because of the anatomical and physiological similarities between its inner ear and that of human (1–4). Its middle ear and Eustachian tube structures are also similar to that of humans and unlike other rodent models it is not susceptible to innate middle ear infections, making it an ideal model organism for otitis media (OM) studies (5–7). The presence of a large cephalid bulla, from which sufficient quantities of middle ear fluids can be withdrawn for microbiological and immunological assessment, is also an advantage. Most interestingly, the chinchilla has a permanently semipatulous Eustachian tube, which makes this tubal organ highly similar to the ‘floppy’ Eustachian tube of childhood (5). The chinchilla is recognized as an ideal model of otitis media and its use has produced critical information regarding the molecular mechanisms of pathogenesis of pneumococcal OM, the immune response to *S. pneumoniae*-induced OM, the efficacy of antimicrobial drugs that target *S. pneumoniae*, and the immunogenicity and protective efficacy of pneumococcal capsular polysaccharide vaccine antigens (8).

Studies using chinchilla to investigate the biology and pathobiology of both bacterial pathogens such as *Haemophilis influenzae*, *Streptococcus pneumoniae*, *Pseudomonas aeruginosa*, *Moraxella catarrhalis*, *Yersinia enterocolitica*, *Listeria monocytogenes* and *Trypanosoma cruzi*, and viral pathogens including adenovirus, Rous sarcoma virus (RSV) and influenza A virus, are also increasing. In addition, the chinchilla is used for studies in renal physiology, cardiac anatomy and pre-clinical assessment of new and improved human vaccine candidates, making it an ideal model to study multiple human diseases and their prevention. Because of its value as a model organism, in 2012, whole genome shotgun sequencing of the genome was completed (9) followed by sequencing of the mitochondrial chromosome (10). The availability of these and other emerging genomic and expression datasets provide researchers with the necessary resources to expand the use of chinchilla as a disease model organism.

### Chinchilla genome

**Figure 1.**
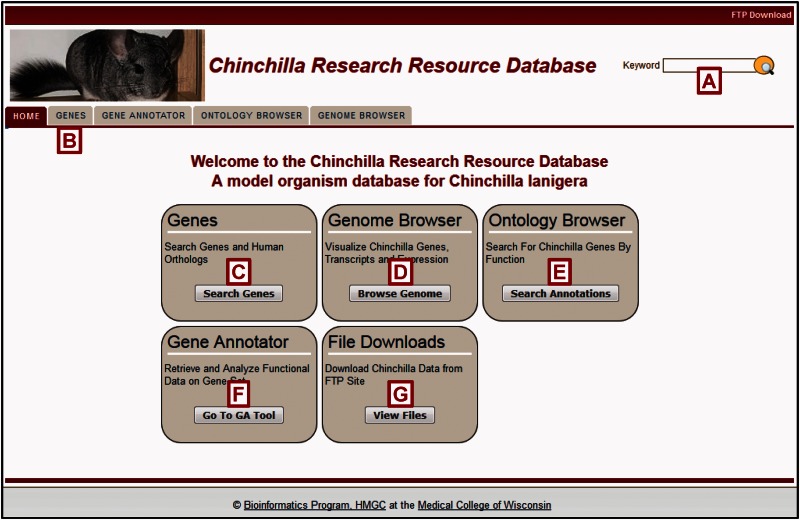
The Chinchilla Research Resource Database (CRRD) Home Page. The CRRD home page gives easy access to keyword (**A**) and gene-specific (**B**, **C**) searches, to the Chinchilla JBrowse Genome Browser (**D**), to the Ontology Browser and Search Tool (**E**), to the Gene Annotator (GA) Tool (**F**) and to the FTP site for bulk data downloads (**G**).

The chinchilla has a diploid genome comprised of 64 chromosomes (32 pairs) of which the X chromosome is the largest (11). Its genome was sequenced at 87X coverage using a whole genome shotgun (WGS) method using the Illumina Hi-Seq technology at the Broad Institute of MIT and Harvard (9) from a sample provided by Dr. Joseph Kerschner of the Medical College of Wisconsin. Using the ALLPATHS v. R40776 approach the genome was assembled into 2839 scaffolds with a total sequence length of 2390.87 MB with the mitochondrion sequence length at 16 580 bases (12). A Gnomon gene annotation analysis conducted at the National Center for Biotechnology Information (NCBI) identified chinchilla genes (13) with additional analysis predicting one-to-one human to chinchilla orthologs (14). Further enhancing these valuable datasets are RNA-Seq expression and transcript datasets for infected and uninfected middle ear mucosa from the Medical College of Wisconsin and for 14 tissues from samples provided by Dr. Lauren Bakaletz's group at the Research Institute at Nationwide Children's Hospital, also available at NCBI (BioProjects PRJNA277957 and PRJNA78823, respectively). This short read RNA-Seq data was used in the genome annotation process and is available in NCBI's Short Read Archive (SRA) for gene and transcript expression studies. The increasing use of chinchilla as a disease model and the availability of critical genomic resources led to the creation of the Chinchilla Research Resource Database (http://crrd.mcw.edu) (Figure 1) to provide investigators easy access to datasets and software tools to enhance their research.

## Chinchilla research resource database

The Chinchilla Research Resource Database was developed to aggregate data from the genome sequencing project and other datasets such as RNA-Seq, and to provide researchers with tools to retrieve and analyze chinchilla data as well as that of human. CRRD was built on existing software architecture previously developed at the Rat Genome Database (15). This approach allows for the re-use of pipelines and interface elements, saving costly development time. The back-end of CRRD is built on J2EE technologies (http://java.sun.com/j2ee/overview.html). Pipelines have been developed in Java 8 and run on a weekly basis to import external data from existing sources such as NCBI’s Gene database (14) and the Rat Genome Database. After the data is processed, it is stored in an Oracle 11 g database that also drives the web site. The Spring framework’s MVC (model-view-controller) architecture streamlines the application web development and provides management of web services (https://projects.spring.io/spring-framework/). The user interface relies heavily on Ajax, Javascript, Angular JS, and CSS (Cascading Style Sheets). Supported browsers include Internet Explorer, Firefox, Chrome and Safari. An FTP server is also available allowing users to perform bulk downloads of gene data, annotations, and raw RNA-Seq data. Where third party software existed which supplied necessary functionality, for example in the case of genome browsers, such software was evaluated and, where advantageous, was incorporated into the infrastructure of the CRRD.

### Genes and orthologs

The Chinchilla Research Resource Database provides access to a comprehensive catalog of over 31 000 chinchilla genes with >75 000 identified transcripts (Table 1) as well as ∼40 000 human genes. Ortholog assignments, computationally derived as part of the NCBI Eukaryotic Genome Annotation Pipeline (16), are updated regularly from the NCBI FTP site, and provide over 16 000 chinchilla-human pairings allowing users to easily query the database for genes of either organism and retrieve the ortholog pairs. NCBI's annotation pipeline predicts orthology based on protein alignments and synteny (17). In keeping with this, essentially all of the chinchilla-human ortholog pairs (16 760/16 774) consist of protein-coding genes in both species.

**Table 1.** Number of genomic elements in Chinchilla

Genomic element	Number
Genes	31 741
Protein-coding genes	21 239
ncRNAs	6233
tRNAs	70
Pseudogenes	4199
Transcripts	75 934
Chinchilla-Human Ortholog pairs	16 774

Breakdown of Genomic Elements for Chinchilla in CRRD. Table 1 lists the number of each type of genomic element, including genes (the total number as well as the number for each gene type), transcripts assigned to those genes, and chinchilla-human ortholog pairs.

**Figure 2.**
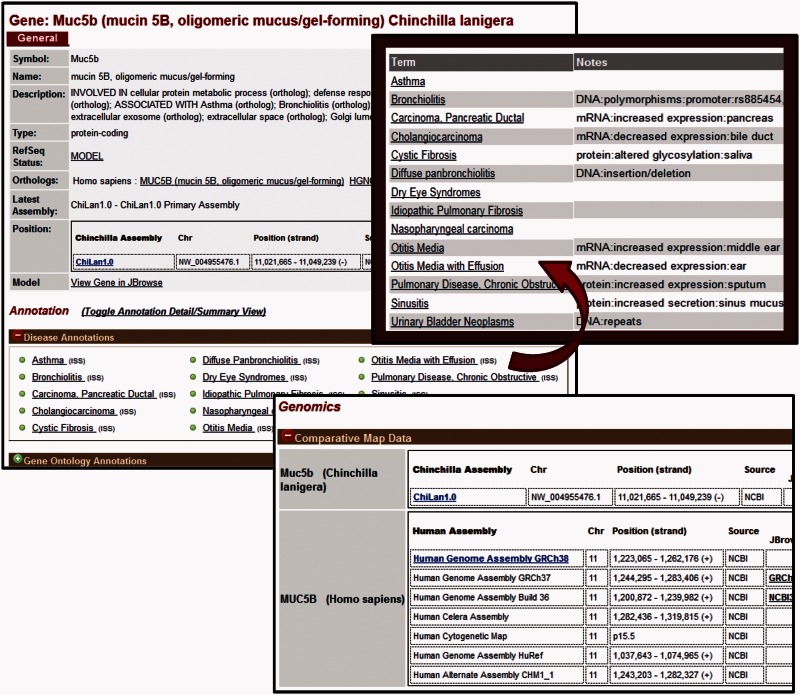
Gene Report Page for Chinchilla. CRRD gene reports display basic information about the gene including the symbol and name of the gene, a description based on the functional annotations for that gene, the gene type as assigned by NCBI and the RefSeq status of the gene. In addition, a link is given to the human ortholog (top left). The Annotations section includes Gene Ontology (GO), Disease and Pathway annotations inferred to chinchilla genes from their human orthologs. When viewing the list of annotated terms, the ‘Toggle Annotation Detail/Summary View’ link can be used to show additional details (top right). Comparative map data includes the scaffold positions for chinchilla genes and positions on multiple assemblies for human (bottom).

Reports are provided for both chinchilla and human genes and include the symbol, description, and a link to the respective ortholog report (Figure 2, top left). Map data for both chinchilla and human genes are also provided. Because the chinchilla genome has not yet been assembled into chromosomes, this consists of the scaffold ID and position for each chinchilla gene. For human, the chromosome and positions on multiple genomic assemblies and on the cytogenetic map are provided for comparative purposes (Figure 2, bottom). Access to multiple identifiers and FASTA sequence files for Genbank and RefSeq nucleotide and protein sequences are available on each gene report. In addition, human gene reports include microRNA target data imported from miRGate (http://mirgate.bioinfo.cnio.es/) (18). Weekly updates from data import pipelines ensure that users have access to the most up to date information available for both chinchilla and human genes.

One of the most valuable aspects of the Chinchilla Research Resource Database is the wealth of functional information made available for chinchilla genes from their human orthologs. Since chinchilla is not one of the most commonly used model organisms such functional information either is not available at all or is extremely limited at databases such as NCBI, UniProtKB and the GO Consortium. Currently, functional annotations to chinchilla genes in CRRD are based on existing annotations to orthologous human genes and are standardized using ontologies appropriate for each functional domain (Table 2). This use of ontologies facilitates data querying, retrieval and analysis. Annotations are presented on each gene report page. Gene Ontology (GO) annotations for biological process, molecular function and cellular component are imported from the GOA Database (19) for the human genes and inferred to the orthologous chinchilla genes. Disease annotations originate from the Rat Genome Database and are based on manual annotations from published literature and imported annotations from resources such as OMIM (20) and ClinVar (21). Manual annotations are created when the results of a study show that an alteration in nucleotide or protein sequence or expression is directly related to a disease or one of its major phenotypes. Such details are noted within each annotation and can be seen by toggling from the default summary view of the annotations to the detail view (Figure 2, top right). An expanded version of the MEDIC Disease Ontology is used to standardize disease associations (22, 23). Pathway annotations also originate from the Rat Genome Database. These are primarily manually curated from the literature (24) or imported from KEGG (25) and are standardized using the Pathway Ontology (26). In addition to these annotations which appear on both chinchilla and human gene reports, annotations for drug–gene and chemical–gene interactions also appear on reports for human genes. These annotations are imported from the Comparative Toxicogenomics Database which uses the ChEBI ontology to standardize annotations (27, 28).

**Table 2.** Chinchilla annotations as of January 2016

Annotation type	Number of annotations
Biological process	147 745
Molecular function	86 569
Cellular component	113 691
Disease	33 515
Pathway	23 922

Number of Annotations Inferred to Chinchilla Genes by Ontology Type. Table 2 shows the number of annotations for each functional domain assigned to chinchilla genes from their human orthologs. The ontologies include the three aspects of the Gene Ontology: Biological Process, Molecular Function and Cellular Component, RGD’s Disease Ontology (RDO, derived from the MEDIC vocabulary) and RGD’s Pathway Ontology.

### Data retrieval and analysis

The Chinchilla Research Resource Database provides multiple methods for retrieving data for single genes or sets of genes. As implied, the *Keyword Search* (Figure 1A) allows users to input gene symbols, names, or keywords related to disease or function and retrieve the gene(s) associated with the input. The more targeted *Gene Search* can be accessed through a tab at the top of each page (Figure 1B) or a box on the home page (Figure 1C). The chinchilla gene search allows gene symbols, names and keywords as input while, because of the completed assembly status of the human genome, filters by chromosome and position on a particular assembly or map type are also available for human gene searches. Genes can also be viewed in their genomic context using the chinchilla Genome Browser (Figure 1D, and see below).

The use of structured ontologies permits easy access to gene sets with similar functional characteristics. The *Ontology Browser* (Figure 1E) offers keyword functions for searches across multiple ontologies as well as the ability to navigate through each ontology tree to reach the desired term.

**Figure 3.**
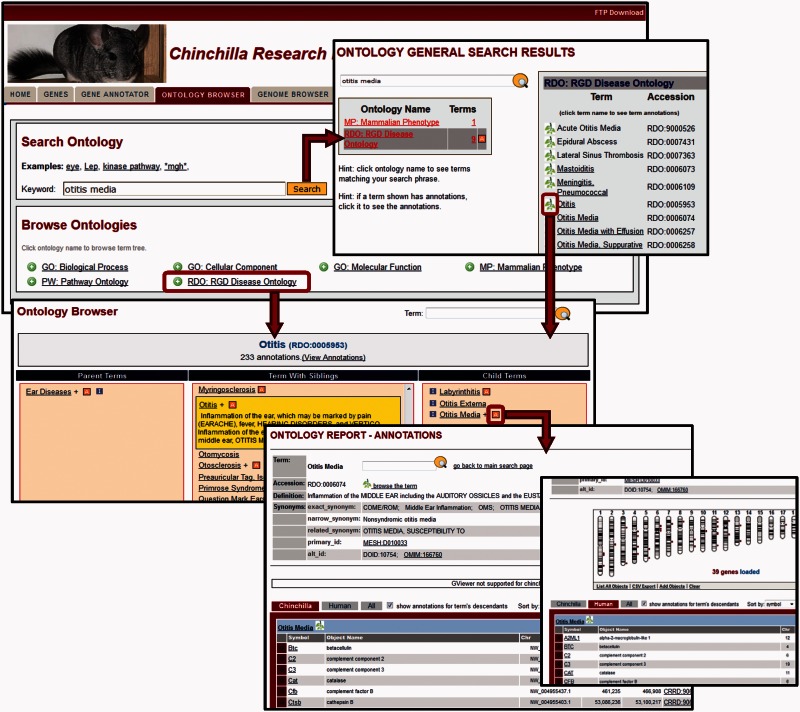
Ontology Browser and Search. Genes with related functional attributes can be retrieved using the CRRD Ontology Browser and Search Tool. A keyword search in the tool will return a scoreboard listing the ontologies with matching terms (top). Select an ontology to see the list of matched terms, to access an ontology report or to begin browsing at a specific term. Alternatively, users can utilize the ontology browser, accessed from the name of the ontology on the main search page or by clicking a leaf icon on the search results page, to navigate up or down the tree to find their term of interest (middle). CRRD Ontology Report pages, accessed via the ‘A’ icon in the browser or the term in the search results, display details of the selected term (bottom left) as well as lists of chinchilla, human or all genes annotated to the selected term and/or its more specific child terms. In addition to the gene list, human genes are graphically displayed at their positions relative to the human karyotype (bottom right) in a whole-genome display on the human-specific results page.

To browse an ontology, the user clicks on the name of the ontology on the ontology search/browse main page (Figure 3, left) and is presented with a three-paned browsing window (Figure 3, middle). Clicking on any term selects that term for display, highlighting it in yellow and showing the term description, synonyms and external identifiers. Selecting a term in the right window moves it to the center pane and presents the user with its more specific child terms to the right. Selecting a term in the left pane again moves that term to the center and presents its more general parent term(s) to the left.

Results from a cross-ontology keyword search (Figure 3, top) are presented as a scoreboard indicating the ontologies with terms matching the input and the number of terms matched in each. A click on the ontology name or term number in the summary scoreboard brings up a list of matching terms. Users can then access the related annotations or further investigate the structure of the related term tree in the same manner as detailed above using options in the details scoreboard. A red ‘A’ in the scoreboard or browser indicates there are annotations to that term (and/or more specific terms in the same branch) within the database. Clicking on this icon or on any term in the details scoreboard will take the user to an ontology report which includes the chinchilla and human genes annotated with the selected term and/or any children terms (Figure 3, bottom). Results include map information and direct links to individual gene reports. For human, a genome-wide view of the annotated human genes at their respective chromosomal positions is supplied. Options are given for downloading the displayed annotation data for both chinchilla and human genes.

**Figure 4.**
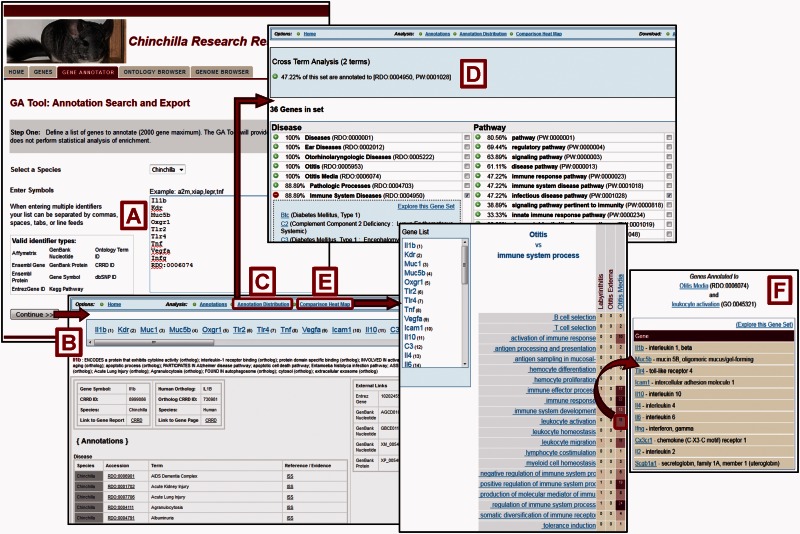
Gene Annotator Tool. The CRRD Gene Annotator (GA) Tool takes a list of gene symbols, CRRD IDs, nucleotide and protein accession numbers, NCBI Gene IDs or Ontology IDs (**A**) and returns the corresponding list of chinchilla or human genes. Results are shown as reports for individual genes (**B**), including functional annotations for the gene and its ortholog, and a list of external database IDs with links to the information at those sites. The Annotation Distribution function (**C**) displays the percentage of genes from the input list which have annotations to categories and/or specific terms in each functional domain (i.e. disease, pathway, biological process, etc.). Select a term, such as ‘Immune System Diseases’, to see the genes from your list that are annotated to that term or to a more specific child term, or select multiple terms to see the genes annotated to all selected terms. Here the terms ‘Immune System Diseases’ and ‘infectious disease pathway’ were selected using the check boxes in the term lists. The Cross Term Analysis results (**D**) show that 47.22% of the list (17/36 genes) are annotated to both. Options are given to explore these gene subsets further. The GA Tool's Comparison Heat Map function (**E**) allows users to browse through two ontologies to see the genes at the intersection of these ontologies at multiple levels. The darker the square in the display, the more genes in the list which have annotations from the ontology branches represented by that column and row. Click a square to see the list of genes represented. The example here shows the list of 11 chinchilla genes which are annotated to both the disease term ‘Otitis Media’ and the Gene Ontology term ‘leukocyte activation’ (**F**).

Once investigators have retrieved a set of genes, the *Gene Annotator* (GA) Tool (Figure 1F) can be used to survey it for commonalities in functional or disease annotations among the genes. Up to 2000 chinchilla or human genes can be uploaded using symbols or CRRD, nucleotide, protein or NCBI Gene identifiers. Users can also generate a gene list using one or more ontology term identifiers (Figure 4A). In addition, for human genes, users can generate the gene list using chromosome number and start and stop positions. Data to be returned for the genes can be customized and any or all ortholog functional annotations can be included. A comprehensive report for each gene is returned with functional annotations and links to sequences and related records in external databases. Reports for all genes in the set can be accessed through links at the top of each page (Figure 4B). The GA Tool does not currently offer an ontology enrichment function, but non-statistical analysis of gene sets can be accomplished through two features: the *Annotation Distribution Tool* and the *Comparison heat map*. The Annotation Distribution Tool (Figure 4C) indicates the percentage of genes in the set associated with particular functional domains such as disease, pathway, biological process, cellular component and molecular function. Clicking the green plus beside a term retrieves the genes from the input set associated with that term and/or its more specific children. Users can also check multiple terms to explore the subset of genes associated with the intersection of these functions (Figure 4D).

The Comparison Heat Map tool (Figure 4E) is another feature that allows investigators to identify and retrieve subsets of genes with similar characteristics. The user can choose the domains represented by each axis in the grid from the ontologies used: disease, pathway, molecular function, biological process and cellular component. Because the ontologies are hierarchical, a click on a higher level term on one of the axes expands that category to show the more specific terms related to it. In this way the user can investigate the number of genes that intersect various levels of classifications. In the example in Figure 4F, the user identified the subset of 11 genes associated with both otitis media and leukocyte activation out of the original 36 genes from the input list. The Gene Annotator also provides data download options for complete datasets or any subset identified in the analysis features.

In addition to downloads from individual searches and analysis results, the *File Downloads *(Figure 1G) feature offers researchers the ability to obtain comprehensive data files. The chinchilla gene file provides symbols, names, descriptions, scaffold IDs, positions and identifiers for NCBI's Gene, Nucleotide, Protein and RefSeq databases for all chinchilla genes. The ortholog file provides symbols and identifiers for chinchilla genes and their human orthologs. Individual annotation files provide gene identifiers and the respective ontology terms and identifiers for each functional domain.

## Chinchilla genome browser

**Figure 5.**
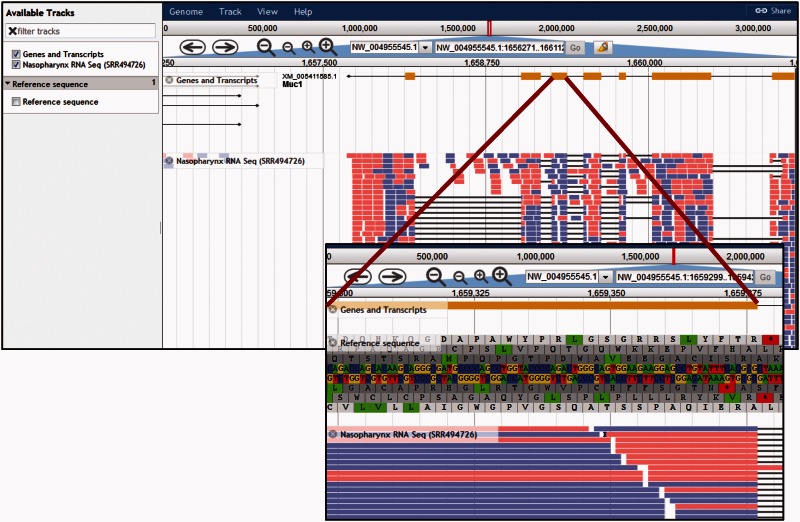
Chinchilla Genome Browser. The ‘Available tracks’ in the CRRD JBrowse genome browser include ‘Genes and Transcripts’, an RNA-Seq BAM alignment for nasopharynx, and the genomic reference sequence. Users can search for a scaffold position or for a gene symbol to navigate to that region of the genome. Navigation buttons allow the user to scroll through the region and zoom in or out to refine their view. The genes and transcripts track shows the intron/exon structure of the gene and, where applicable, any predicted splice variants. The RNA-Seq track displays the BAM alignments for short read RNA sequences from chinchilla nasopharynx. When zoomed in sufficiently, the ‘Reference Sequence’ track shows the reference DNA sequence and the corresponding predicted amino acid sequences in all six reading frames (insert).

Although the chinchilla genomic sequence has only been assembled to the scaffold level, CRRD provides a genome browser for viewing genes in their genomic context (Figures 1D and 5). The browser is an instance of the JBrowse browser from the Generic Model Organism Database (29). Currently three tracks are available: the CRRD Genes and Transcripts track, an RNA-Seq track showing BAM alignments for nasopharyngeal mucosa (SRA Run ID SRR494726; BioSample SAMN00991504), and the reference sequence track displaying the genomic nucleotide sequence and amino acid sequences in all six reading frames (Figure 5, inset). The dropdown list which usually contains chromosome numbers in the header section contains the NW identifiers of the 300 longest scaffolds out of 2839. If the scaffold position is known, this can be entered into the search box as ‘NW_ID:start..stop’. Alternatively, a gene symbol can be entered to navigate to that region of the genome. The CRRD Genes and Transcripts track shows the intron/exon structures of transcripts predicted by NCBI's Gnomon gene annotation analysis. Clicking a gene opens a popup with additional information, including links for downloading the FASTA sequence of the exons.

Because of the large volume of data contained in an RNA-Seq BAM alignment, individual segment alignments are only visible when the view is sufficiently zoomed in. At this level of resolution, individual segments are displayed as color-coded blocks, red when the alignment is to the positive strand and blue when aligned to the negative strand. Click any block to show a popup displaying data extracted from the source BAM alignment file, including the sequence, match type, overall mapping quality score and single nucleotide quality scores.

## Future directions

Plans are underway to expand this resource to accommodate emerging datasets, among which are the inclusion of ortholog data from additional model organisms, including rat and mouse. The addition of these orthologs will also bring in the extensive mouse phenotype (MP) dataset and the growing body of rat phenotype data from inbred, outbred and genome-edited animals. A two-pronged approach to gene and phenotype annotation will encompass extraction of data from existing literature by expert curators as well as a community annotation project whereby researchers can contribute annotations based on their ongoing research. This approach will result in the addition of critical chinchilla-specific phenotype measurement data, host-pathogen interaction data covering a variety of pathogenic organisms, and functional annotations (GO, disease and pathway) for genes with experimental evidence in the chinchilla. Additional software tools to mine and analyze these data types will be incorporated. Finally, the chinchilla JBrowse will be expanded to incorporate all currently available datasets, as well as future experimental results.

## Discussion and conclusion

The chinchilla is a valuable animal model for a number of diseases of the inner and middle ear and others involving multiple human viral and bacterial pathogens and polymicrobial infections. The release of the chinchilla genome and emerging transcriptome datasets has increased the value of this model organism for disease studies, and creation of a full chromosome-level assembly out of the existing scaffolds would be another important step in this direction. A full assembly would provide the material necessary to improve cross-organism studies and thus improve researchers’ ability to identify genomic elements important to disease processes and resolution. The Chinchilla Research Resource Database is an important resource that allows researchers easy access to the chinchilla gene catalog and the respective human ortholog set. The comprehensive functional information on disease associations, pathways, biological processes and molecular functions and the related analysis tools create a rich research platform that will help inform the design of investigations to unlock the mechanisms of disease and the pathways to their resolution and/or prevention. Expansion of this resource as outlined will further enhance the value of the Chinchilla Research Resource Database.

## Funding

This work was supported by a grant from the Medical College of Wisconsin (MCW) entitled ‘MCW Dean’s Program Development: Chinchilla Genome’

*Conflict of interest*. None declared.
